# Applications of extraembryonic tissue-derived cells in vascular tissue regeneration

**DOI:** 10.1186/s13287-024-03784-3

**Published:** 2024-07-09

**Authors:** Mehdi Amiri Goushki, Zahra Kharat, Mousa Kehtari, Alireza Naderi Sohi, Hana Hanaee Ahvaz, Iman Rad, Simzar HosseinZadeh, Fatemeh Kouhkan, Mahboubeh Kabiri

**Affiliations:** 1 Department of Life Science Engineering, Faculty of New Sciences & Technologies, University of Tehran, Tehran, 14395-1561 Iran; 2 Department of Biotechnology, College of Science, University of Tehran, Tehran, 14155-6455 Iran; 3 School of Biology, College of Sciences, University of Tehran, Tehran, 1417614411 Iran; 4 National Institute of Genetic Engineering and Biotechnology, Tehran, 1497716316 Iran; 5 Stem Cell Technology Research Center, Tehran, 15856-36473 Iran; 6 Department of Tissue Engineering and Regenerative Medicine, School of Advanced Technologies in Medicine, Shahid Beheshti University of Medical Sciences, Tehran, Iran

**Keywords:** Vascular tissue regeneration, Extraembryonic tissue-derived cells, perinatal tissues, Stem cells

## Abstract

Vascular tissue engineering is a promising approach for regenerating damaged blood vessels and developing new therapeutic approaches for heart disease treatment. To date, different sources of cells have been recognized that offer assistance within the recovery of heart supply routes and veins with distinctive capacities and are compelling for heart regeneration. However, some challenges still remain that need to be overcome to establish the full potential application of these cells. In this paper, we review the different cell sources used for vascular tissue engineering, focusing on extraembryonic tissue-derived cells (ESCs), and elucidate their roles in cardiovascular disease. In addition, we highlight the intricate interplay between mechanical and biochemical factors in regulating mesenchymal stem cell (MSC) differentiation, offering insights into optimizing their application in vascular tissues.

## Introduction

Despite progressive advances in pharmacological and surgical therapy, cardiovascular diseases remain the leading cause of mortality worldwide [1]. These diseases typically involve the buildup of fatty deposits within arteries and an increased risk of blood clot formation. The increasing demand for organ transplants and the limited supply of donor organs have resulted in organ shortage crises. Conventional synthetic vascular grafts are incompetent at remodeling and have limited patency, particularly at smaller diameters. This is where tissue engineering has emerged as a promising solution. By combining bioactive cells with biodegradable scaffolds, tissue-engineered vascular grafts can be developed, offering the advantages of growth and self-healing capacity. These features have the potential to overcome the limitations of conventional treatment approaches [2].

Presently, the treatment of cardiovascular diseases primarily involves a combination of dietary and lifestyle modifications, medical intervention, and surgical procedures, with surgery typically reserved for advanced cases. Surgical interventions include endovascular techniques such as angioplasty and stent placement to widen or clear narrowed blood vessels. Vascular bypass grafting is another surgical method that is employed to circumvent damaged or blocked vessels. This procedure commonly utilizes autologous arteries or veins. However, due to the limited availability of arteries and severe complications associated with their removal, the saphenous vein is more frequently used as an autograft vessel than the arteries [3]. Nevertheless, the use of autologous vessels also presents certain drawbacks, such as low quality and site morbidity stemming from the extraction process [4].

Tissue engineering represents a rapidly evolving field with the ambitious goal of creating functional tissues and organs through the integration of cells, biomaterials, and biochemical factors. One of the most promising avenues in tissue engineering is stem cell-based therapy. Stem cells possess the remarkable capacity to differentiate into diverse cell types, making them ideal candidates for regenerating damaged or diseased tissues. The use of stem cells in tissue engineering offers several advantages over traditional therapies. First, stem cells can be obtained from various sources, including bone marrow, adipose tissue, or embryonic tissue. This versatility allows for personalized treatment strategies and reduces the risk of immune rejection. Second, stem cells can be cultivated in vitro, enabling the generation of substantial quantities of cells needed for transplantation. This scalability makes it possible to treat a wide range of patients with different tissue defects [5].

Stem cell-based therapy has shown promising results in the treatment of a diverse range of conditions, including heart disease, spinal cord injury, and liver failure. For example, in the realm of cardiac tissue engineering, stem cells can differentiate into cardiomyocytes, specialized heart muscle cells, and subsequently be seeded onto a scaffold to create functional heart muscle patches. These engineered patches have the potential to be transplanted into patients with damaged cardiac tissues, offering a promising avenue for enhancing overall cardiac function and potentially restoring heart health [6].

Among the different sources of stem cells, perinatal stem cells have emerged as a promising source for vascular regeneration due to their unique characteristics and potential applications, including lack of need for invasive procedures to obtain them, high degree of plasticity, immunomodulatory properties, low risk of tumorigenesis, younger and healthier cells compared to other adult stem cells, and angiogenic potential [7]. These valuable features make perinatal stem cells ideal candidates for regenerating damaged blood vessels and developing new therapeutic approaches for cardiovascular diseases. One of the key advantages of perinatal stem cells is their easy accessibility. Unlike other sources, such as bone marrow or adipose tissue, perinatal stem cells can be obtained noninvasively and do not pose any risk to the mother or baby during childbirth. Such readiness for availability and ethical advantages have contributed to their increasing popularity in research and clinical settings. Furthermore, perinatal stem cells exhibit high proliferative capacity and immunomodulatory properties [8]. They can be expanded in culture while maintaining their differentiation potential, making them an abundant source for large-scale production of vascular tissues [9]. Perinatal stem cells exhibit a high degree of plasticity, meaning that they can differentiate into various cardiovascular cell types, including endothelial cells, smooth muscle cells, and fibroblasts, which are crucial for vascular tissue engineering. This versatility allows them to contribute to the formation of functional blood vessels and vascular networks. Additionally, these cells possess immunosuppressive capabilities that allow them to evade immune rejection when transplanted into a recipient [7]. However, further research is needed to understand their long-term effects, optimize their differentiation protocols, and enhance their therapeutic efficacy. This paper reviews the different cell sources for vascular tissue engineering, focusing on extraembryonic tissue-derived cells.

## Functional requirements for vascular grafts

**Fig. 1:**
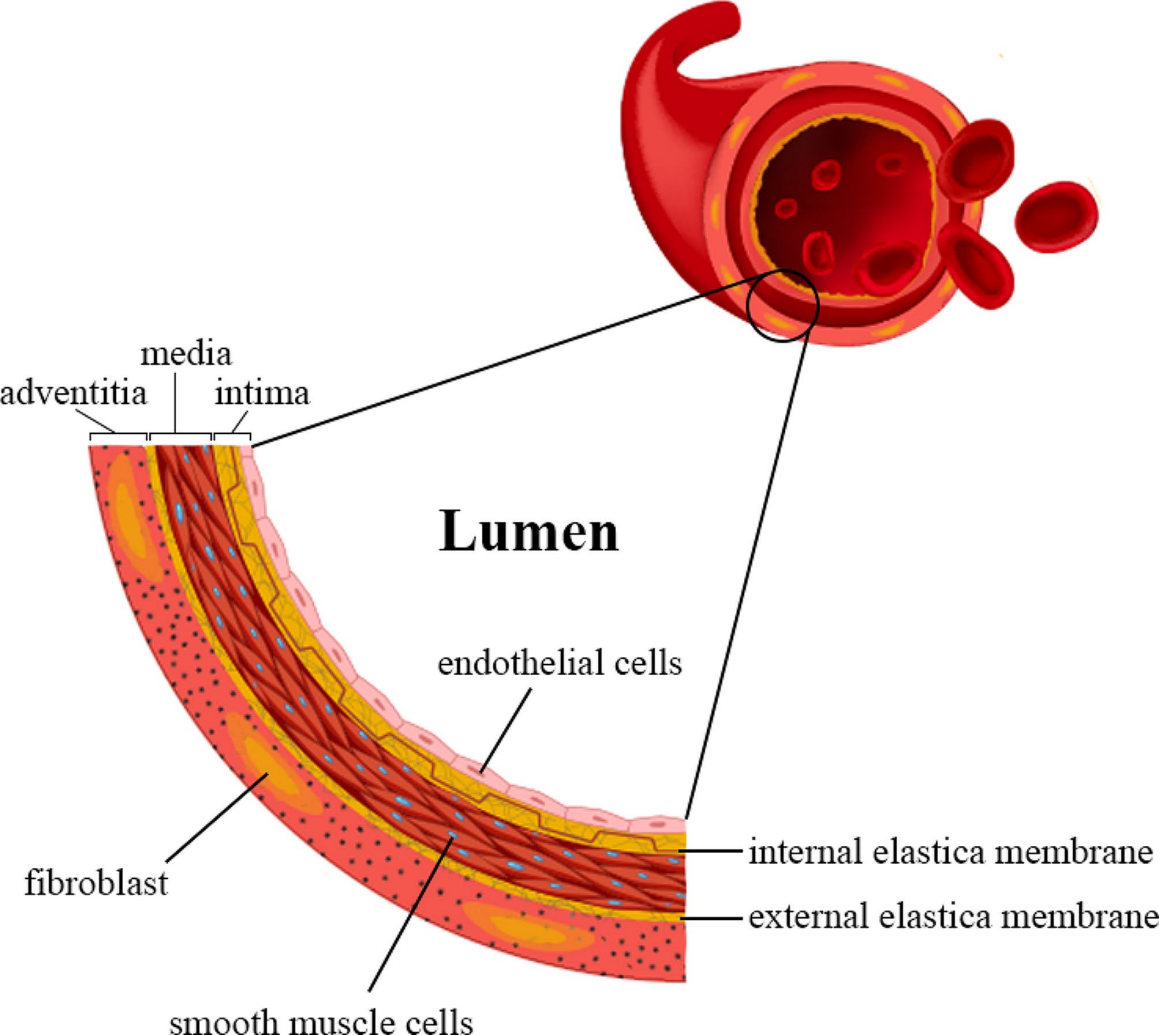
Basic structural characteristic of a blood vessel

Blood vessel walls typically consist of three main layers. The innermost layer adjacent to the lumen, known as the “tunica intima”, is composed of endothelial cells (ECs) and intimal smooth muscle cells (SMCs). The middle layer, called the “tunica media”, is composed of medial smooth muscle cells. Eventually, the outermost layer, called the “tunica adventitia”, comprises fibroblasts and extracellular matrix (ECM) components as well as microvessels (vasa vasorum), particularly in large blood vessels [10, 11] (Fig. 1). Although autologous vascular transplantation minimizes immune responses, the high occurrence of occlusion and the need for multiple surgical procedures have restricted the effectiveness of autologous replacement approaches [12].

Vascular graft tissue engineering constitutes a crucial aspect of tissue engineering with the objective of regenerating or generating new blood vessels with the help of bioactive and biocompatible materials [13].

Synthetic vascular grafts serve as substitutes for autologous vessels under certain conditions. In the long term, these grafts have shown satisfactory outcomes when used in large-diameter arteries (> 8 mm); however, their applicability in small-diameter vessels (< 6 mm) is limited due to unfavorable patency rates [14]. To address this limitation, attempts have been made to enhance the performance of synthetic grafts. One such approach involves the seeding of autologous endothelial cells onto the luminal surface of synthetic graft vessels, which has been shown to improve patency rates. Nevertheless, even with these improvements, the performance of synthetic grafts has not surpassed that of autologous vessels [15].

The first engineered blood vessel structures were produced in the mid-1980s by Weinberg and Bell [16]. Since then, various methods have been adopted to produce similar structures. In general, scaffold-based production methods can be classified into three categories: synthetic polymers, biopolymers, and biodegradable polymers [17].

Vascular tissue engineering typically entails meeting four fundamental requirements: suitable mechanical properties, blood compatibility, endothelial support, and biodegradability.

### Mechanical properties

Tissue-engineered vascular grafts must have specific mechanical characteristics. These include compliance, which allows seamless integration with adjacent vessels, and the appropriate mechanical strength and elasticity to withstand blood pressure without deformation [18].

### Blood compatibility

Blood compatibility refers to the ability of a material or device to interact with blood without causing adverse reactions [19]. Like other blood-related devices, tissue engineering vascular grafts (TEVGs) share similar mechanisms related to thrombosis; however, there are various antithrombotic strategies for the modification of TEVGs, including the use of antithrombotic agents, platelet inhibitors, or fibrinolysis agents. One effective approach involves surface endothelialization because the vascular endothelium on the inner surface of blood vessels is inherently compatible with blood. Another method is surface passivation, which prevents external surfaces from coming into contact with blood and can be achieved through the use of zwitterionic or hydrophilic polymers [18].

### Endothelial friendliness

The primary goal of vascular graft tissue engineering is to promote neovascularization and the formation of new blood vessels, which mainly depend on the secretion of ECM by adherent endothelial cells. Therefore, an ideally compatible TEVG should guarantee the adhesion of endothelial cells and provide a conducive microenvironment for cell proliferation supported by the presence of growth factors and bioactive molecules [18].

Several strategies can enhance endothelium friendliness in TEVGs. Some growth factors can be directly incorporated into the polymer solution before electrospinning [20] or attached to heparin through covalent bonding [21]. Polydopamine, known for its anchoring properties, can also be utilized to coat the graft surface with growth factors [22].

However, it is essential to consider the relatively short half-life of growth factors, which can lead to their inactivity. To address this limitation, sustainable release methods have been developed to ensure prolonged release. Another alternative method involves modifying endothelial cells to secrete growth factors. By introducing pDNA to produce growth factors in transfected endothelial cells, both cell viability and ECM production can be significantly increased [23].

### Biodegradability

In vascular graft tissue engineering, the choice of polymer is crucial, and it should ideally be biodegradable to reduce concerns related to immunogenicity and thrombus formation. The biodegradation of the polymer should align with the pace of new blood vessel formation. Premature or delayed degradation may hinder blood vessel formation and exacerbate immunogenicity concerns, respectively. Polycaprolactone (PCL), poly glycolic acid (PGA), and Polylactic acid (PLA) are among the common biodegradable polyesters used in this context. These polymers offer controlled degradation properties, allowing for synchronized tissue regeneration and polymer breakdown [24].

Since any deficiency in these aspects causes a disturbance in the neovascularization process, all four requirements must be coordinated and work in harmony [18].

## Cell sourcing in vascular tissue engineering

Selecting the right cell source is a crucial step in achieving success in tissue engineering. Considerations should encompass vital characteristics such as the rate of cell survival, proliferation potential, differentiation capabilities, ability to integrate into the host tissue, and ease of accessing these cells (Table 1).

### Autologous vascular cells

The term “autologous vascular cells” typically refers to endothelial cells and vascular smooth muscle cells sourced directly from the patient’s own body, making them very compatible in terms of immunity. However, the practical application of primary autologous cells presents certain challenges, prompting researchers to explore alternative approaches, such as deriving ECs and vascular smooth muscle cells (VSMCs) from stem cells. These challenges include limitations in cell quantity due to donor age, as well as issues related to poor proliferation and regeneration capacity [25].

### Mesenchymal stem cells (MSCs)

MSCs, also referred to as mesenchymal stromal cells, are the most promising candidates for vascular tissue engineering. These cells are particularly appealing because of their ease of isolation, rapid expansion rate, immune evasion [26] and potential for differentiation into mesodermal lineages, including osteocytes, chondrocytes, adipocytes, and even nonmesodermal lineages, such as neural cells [27], hepatocytes [28] and pneumocytes [29]. The ability of MSCs to differentiate toward vascular lineages, including ECs and SMCs, coupled with their secretion of paracrine factors, position them as valuable contributors to vascular regeneration [30]. This versatility and regenerative potential make MSCs a promising avenue for advancing vascular tissue engineering efforts. In the following section, we will highlight three key attributes of MSCs that make them an appealing cell source for VTE treatment.

### Progenitor cells

Progenitor cells are descendants of stem cells and require further differentiation to achieve specialization in specific cell types. These cells exhibit a distinct propensity to mature into their intended cell lineage. Progenitor cells are found throughout the body, obviating the necessity of isolating them from vessels. According to previous studies, progenitor cells such as endothelial progenitor cells (EPCs), smooth muscle progenitor cells (SMPCs), and pericytes are effective in vascular repair. During inflammation and shear stress, these cells are activated and begin to differentiate into different types of vascular cells. They also improve vascularization and angiogenesis [31–33]. However, an inherent challenge arises in their application, particularly in the elderly population, as the quantity of these cells and their differentiation potential diminish with age [33].

### Emergence of Induced Pluripotent Stem cells (iPSCs)

iPSCs, a breakthrough in stem cell biology pioneered by Yamanaka et al., who earned a Nobel Prize in 2012, can differentiate into any cell type, offering vast potential for personalized medicine. Derived from accessible tissues such as skin or blood, iPSCs circumvent ethical issues and reduce immunogenic risks, representing promising advancements in tissue engineering, such as the production of vascular grafts. However, their use is limited by their potential for tumorigenicity, the presence of mitochondrial DNA mutations, and the complex, costly process of cell reprogramming and standardization [25, 34].

**Table 1:** Cell sourcing in vascular tissue engineering

Cell Source	Characteristics	Advantages	Challenges	Reference
Autologous Vascular Cells	Derived from the patient’s own vascular tissue	• High Immunocompatibility • No need for external cell sources • Can be used in autologous transplants.	• Limited availability and quantity, especially for small-diameter grafts. • Invasive biopsy procedure with potential complications.	[25]
Mesenchymal Stem Cells (MSCs)	Highly versatile, immune-evasive, and expansive	• Easy isolation from various sources (e.g., bone marrow, adipose tissue, and umbilical cord). • High expansion rate. • Differentiation potential into various cell types, including endothelial and smooth muscle cells. • Abundance • unaffected by age. • Immune compatible when derived from the patient. • Paracrine effects contribute to vascular repair.	• Potential for chromosomal rearrangements and tumorigenicity during differentiation. • Risk of mitochondrial DNA mutations. • Time-consuming and costly production and standardization process. • Complexities in optimizing differentiation methods.	[26, 27, 30]
Progenitor Cells	Descendants of stem cells need further differentiation	• Abundant throughout the body. • Can be isolated from vessels. • Immunocompatibility is an issue, particularly in elderly individuals.	• Limited differentiation potential in the aging population. • Challenges in harvesting and culturing.	[31, 32]
Induced Pluripotent Stem Cells (iPSCs)	Reprogrammed somatic cells with versatile potential	• Can be derived from easily accessible tissues (e.g., skin or peripheral blood). • Minimal immunogenic reactions when derived from the patient. • Potential for unlimited cell source. • Ability to differentiate into various cell types, including smooth muscle and endothelial cells for vascular grafts.	• Potential for chromosomal rearrangements and tumorigenicity during reprogramming. • Risk of mitochondrial DNA mutations. • Time-consuming and costly production and standardization process.	[34]

## Extraembryonic perinatal tissues: new putative sources of stem cells

Perinatal stem cells (PSCs) are a remarkable category of stem cells derived from tissues that develop from the 20th week of pregnancy through the 4th week after birth [35]. While adult stem cells from various tissues hold promise as potential sources for cell therapy applications, certain challenges, including ease of isolation, pluripotency, self-renewal, and ethical considerations, narrow the range of viable tissue sources for cell isolation. Among the commonly sought-after tissues for stem cell extraction, such as adipose tissue and bone marrow, perinatal tissues such as the placenta, amnion, and chorion have proven to be rich sources of stem cells. The stem cells obtained from these perinatal tissues possess highly desirable characteristics, such as ease of access, abundance, noninvasive extraction methods, minimal ethical problems, and reduced immunologic compatibility problems [36]. Stem cells isolated from perinatal tissue can be broadly categorized into two main groups: hematopoietic stem cells (HSCs) and fetal MSCs. These cells are placed between embryonic and adult cells in terms of their stemness abilities [37].

### Hematopoietic stem cells (HSCs)

HSCs, characterized by their multipotent nature, serve as precursors for various types of blood cells. HSCs isolated from bone marrow have a long history of use in the treatment of hematologic malignancies. However, a significant milestone in the field of stem cell therapy occurred in 1988 when umbilical cord blood cells were first successfully transplanted to treat genetic blood disorders. The patient was a young boy with Fanconi anemia who received cord blood cells, marking a pivotal moment in the therapeutic potential of cord blood stem cells. After this success, subsequent research revealed that cord blood cells exhibit remarkable differentiation capabilities, giving rise to erythroid, myeloid, and lymphoid cell lineages [37].

### Fetal mesenchymal stem cells

In the past, the prevailing belief was that the main source of MSCs was the bone marrow. However, it has become evident that the number of MSCs in the bone marrow is relatively low, and their regenerative capacity diminishes after passage 10–12 [38]. Therefore, bone marrow-derived MSCs present limitations that restrict their suitability for cell therapy purposes. In contrast, fetal MSCs isolated from perinatal tissue, including the umbilical cord, umbilical cord blood, placental blood, and placenta, do not have these limitations and can be regarded as a valuable alternative for cell therapy purposes [39]. Fetal MSCs can differentiate into three cardiac lineages: cardiomyocytes, endothelial cells, and smooth muscle cells. Additionally, these cells are able to form capillary structures on Matrigel, while mesenchymal cells isolated from adult sources (bone marrow and adipose tissue) cannot. Therefore, the selection of MSCs from fetal sources, such as the umbilical cord or amniotic membrane/fluid, can be an attractive source of young cells for autologous cell transplantation [40]. In the following sections, the use of MSCs from perinatal tissues is discussed in more detail.

## Stem cells in perinatal tissues

**Fig. 2:**
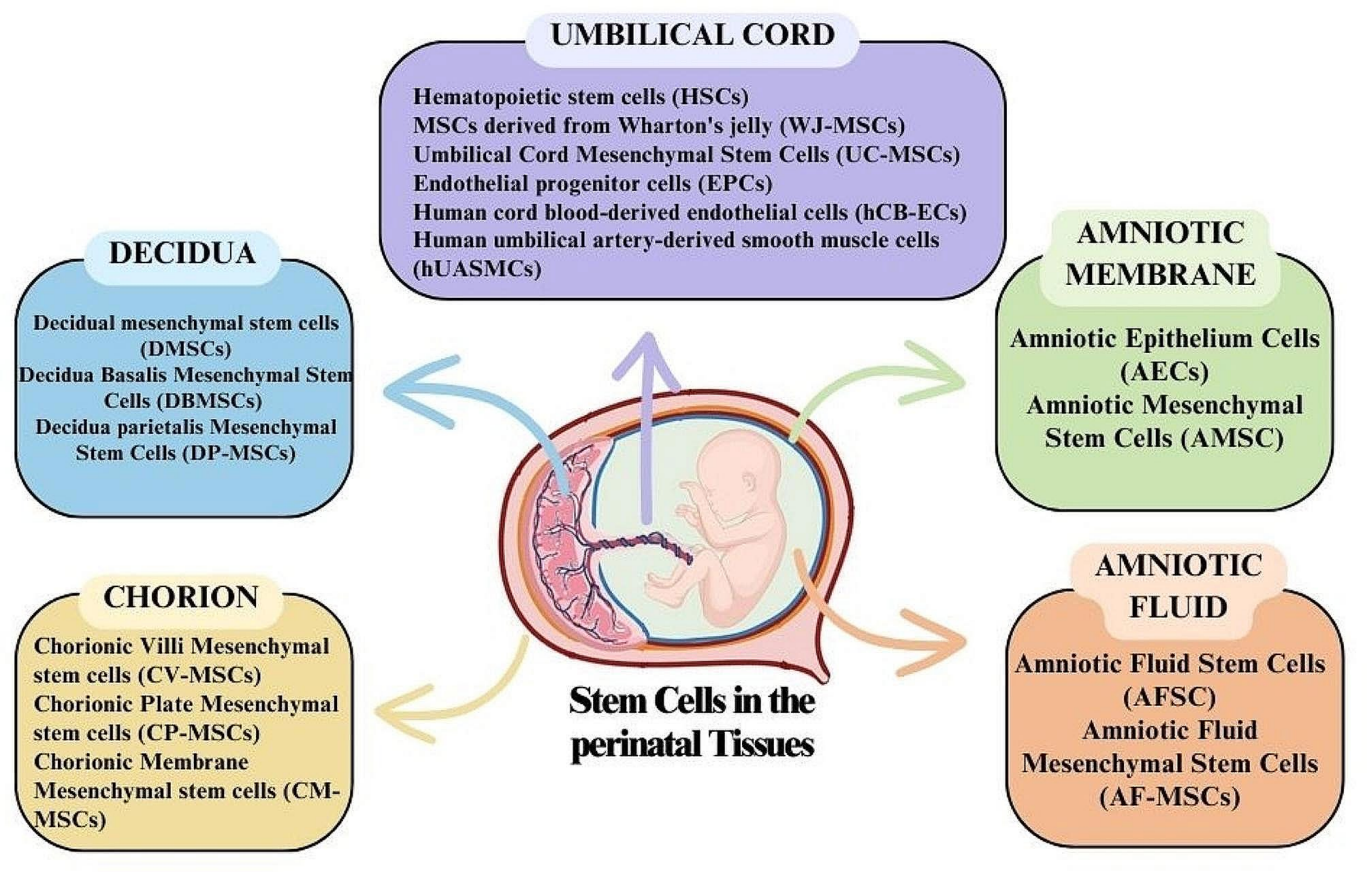
Various sources of extraembryonic tissue-derived stem cells used in vascular tissue engineering

The use of umbilical cord blood in transplantation has a history spanning over 30 years. In addition to cord blood, the placenta and fetal annexes, including the amniotic membrane, chorionic villi, chorionic plate, Wharton jelly of the umbilical cord, decidua, and amniotic fluid, are rich repositories of diverse stem cell types. Among these, one can find trophoblast epithelial stem cells and mesenchymal stromal cells, further underscoring the abundant and varied stem cell resources within perinatal tissues [7]. The exploration of perinatal stem cell sources offers substantial promise for a broad spectrum of therapeutic applications, notably in the field of cardiovascular diseases. In the subsequent sections, we will delve into the specific applications of these cell sources and elucidate their roles in vascular tissue engineering. (Fig. 2).

### Amniotic membrane

The amniotic membrane (AM) is the innermost membrane lining the fetal placenta and consists of five distinct layers, including the epithelial layer, which directly interfaces with amniotic fluid and is composed of amniotic epithelium cells (AECs). Beneath the epithelial layer lies the basement membrane, followed by the compact layer. The fibroblast layer is situated beneath the compact layer, and the spongy layer completes the structure. The basic function of the amniotic membrane is to provide a protective and supportive environment, shielding the developing embryo from external threats, potential infections, and harmful toxins [41]. The membrane has emerged as a suitable candidate for clinical applications, serving as a scaffold for tissue engineering or a carrier for the delivery of various cell types. AM possesses unique biological features, including low immunogenicity, antibacterial capacity, immunomodulation, antiscaring, anti-inflammatory properties, and hemocompatibility, as well as antiangiogenic and proapoptotic qualities, all without presenting ethical challenges [42–44]. Amniotic epithelial and mesenchymal cells isolated from AMs exhibit stem cell properties and express stemness markers such as Oct3/4, Sox2, Klf4, Nanog, TRA1-60, TA1-81, and SSEA4. Notably, these cells do not display tumorigenic characteristics after transplantation, largely due to the loss of their telomerase activity. Both AECs and amniotic mesenchymal stem cells (AMSCs) possess unique properties, including low immunogenicity, anti-proliferative effects on immune cells, anti-inflammatory properties, proliferation capability, and multipotent differentiation potential into cells with mesodermal, ectodermal, and endodermal lineages, and there is no need for invasive techniques to harvest them because they are discarded after birth [42, 43].

Amnion-derived epithelial and mesenchymal cells inhibit tumor growth and invasion through three pathways: the induction of B and T lymphocyte apoptosis through the secretion of soluble factors, including TNF-α, FasL, TRAIL, TGF-β, and macrophage migration-inhibitory factors; the stimulation of cell cycle arrest; and the inhibition of angiogenesis. Therefore, a new line of research has been initiated with a focus on the antiproliferative effects of amniotic epithelial and mesenchymal cells on tumor growth in the last decade [42].

AECs can integrate into the tumor vascular lumen, differentiate into ECs or smooth muscle cells, reduce dysregulated tumor angiogenesis, enhance vascular perfusion, and subsequently, induce the cytotoxic effects of cisplatin. Since the vascular structure is not repaired because of the interconnected and unstable molecular pathways of the tumor, the vasculature can only be rebuilt within a limited time. Evaluation of the multipotential stem cell features of AECs to monitor the vascular formation abilities of AECs in vitro and in vivo under different conditions confirmed that AECs differentiated into tumor vascular endotheliocytes or pericytes and enhanced tumor vascular integrity to more efficiently kill the tumor during treatment, providing a new approach for rebuilding tumor vascularity [45].

### Amniotic fluid

Amniotic fluid (AF), found within the amnion cavity surrounding the developing fetus, plays a vital role in fetal protection and nourishment during embryogenesis. This fluid consists of water, various chemical substances, and an abundant supply of stem cells (especially amniotic fluid stem cells (AFSCs) and amniotic fluid mesenchymal stem cells (AF-MSCs)). These stem cells exhibit essential stem cell markers, including Oct-4, c-Myc, Sox2, Nanog, and SSEA3, as well as high levels of several MSC markers, including CD29, CD44, CD73, CD90, CD105, CXCR4, stromal cell-derived factor 1 receptor (SDF-1), CD146, CD166, and CD184. Importantly, AFSCs can be safely collected without ethical concerns, either during the second or third trimester of pregnancy or even at later stages. Their unique characteristics combined with their stem cell properties and low immunogenicity make AFSCs highly suitable for various applications in the field of tissue engineering [46].

The pluripotency of AFSCs falls between that of ASCs and ESCs. Amniotic fluid stem cells exhibit remarkable proliferation capacity, with significant growth observed within just 36 h. Importantly, AFSCs maintain stable telomerase activity and a normal karyotype even after undergoing 250 in vitro amplification cycles [47].

Human AFSCs can promote vascular development and increase vessel length, area, and thickness in cocultures. These cells can differentiate into ECs, known as AFSC-derived ECs (AFSC-ECs), which have endothelial-like cell functions and can form cohesive vascular networks. A study conducted in 2015 showed that coculturing AFSCs with AFSCs and ECs within a fibrin/PEG matrix had a synergistic effect on various network parameters. The results revealed that the development of robust vessels relied on the presence of both AFSCs-ECs and AFSCs, each contributing to their vasculogenic and perivascular potential, respectively. In addition, fibrin/PEG hydrogels not only promoted capillary-like network formation but also offered advantages in terms of biocompatibility, mechanical stability, and vasculogenesis [48].

In 2016, Zhang et al. investigated the impact of glial cell line-derived neurotrophic factor (GDNF) on the differentiation of AFSCs into vascular endothelial-like cells. GDNF, known for its role as a neurotrophic factor of dopaminergic neurons, also acts as a tissue morphogen that enhances the differentiation of stem cells. The results indicated that AFSCs express EC markers such as vWF and CD31 after one week of differentiation and can be applied at vascular injury sites to promote angiogenesis. While GDNF improved the capacity of AFSCs to differentiate, these cells did not display the complete vascular morphology of mature ECs [47].

Human AFSCs can be induced to differentiate into vascular ECs by overexpressing the Ets transcription factors (TFs) Etv2, Fli1, and Erg. These converted amniotic cells successfully acquired an EC-like immunophenotype; however, their functional performance in EC assays was inferior to that of fully mature ECs. Sox17 expression is crucial for the proper functioning of ECs in vascular engraftment. Whereas Ets transcription factors help distinguish EC-like cells from nonvascular amniotic cells, Sox17 increases morphogenesis gene expression and facilitates the integration of transplanted converted cells into injured blood vessels. Therefore, the regulation of the Sox17 gene is essential for the generation of long-lasting, engraftable, and stable ECs following initial EC gene induction by Ets [49].

In addition, AF-MSCs have shown superior potential compared to BM-MSCs because they can differentiate into multiple cell lineages representing all three embryonic germ layers, such as chondrocytes, ECs, hepatocytes, and neurons, highlighting their remarkable versatility [50]. The characteristics of amniotic fluid stem cells (AFSCs), amniotic ECs (AECs), and AMSCs are compared in Table 2, and an overview of research in the field of vascular tissue engineering involving these cells is shown in Table 3.

**Table 2:** Comparison of the features of amniotic fluid stem cells (AFSCs), amniotic ECs (AECs), and amniotic mesenchymal stem cells (AMSCs) based on the following information:

Feature	AFSC	AFMSC	AEC	AMSC
Stem Cell Markers	Oct-4, c-Myc, Sox2, Nanog, SSEA3	CD117, CD44, CD105, CD90, Oct-4	Oct3/4, Sox2, Klf4, Nanog, TRA1-60, TA1-81, SSEA4	CD90, CD44, CD73, CD105, CXCR4, CD146, CD166, CD184
Differentiation Potential	Mesodermal, ectodermal, endodermal lineages	multiple cell lineages of three embryonic germ layers, such as chondrocytes, endothelial cells, hepatocytes and neurons	Endothelial, Smooth Muscle, Mesodermal	Multiple lineages (mesodermal)
Proliferation Capacity	High	High	High	High
Telomerase Activity	Stable	Stable	Loss of activity	Stable
Immunogenicity	Low	Low	Low	Low
Ethical Concerns	No ethical concerns	No ethical concerns	No ethical concerns	No ethical concerns
Vascular Differentiation	Can differentiate into ECs (AFSC-EC)	Can differentiate into endothelial-like cells	Yes	Limited vascular morphology
Impact of GDNF	Promotes differentiation into endothelial-like cells	-	-	-
Ets Transcription Factors Impact	Acquire EC-like immunophenotype	Acquire fibroblast-like morphology	Acquire EC-like immunophenotype	Acquire EC-like immunophenotype
Sox17 Expression Impact	Essential for vascular engraftment	-	Essential for vascular engraftment	Essential for vascular engraftment
Preclinical Applications	Suitable for cardiovascular patches and vascular grafts	Cardiac malformation [88]	Suitable for cardiovascular patches and vascular grafts	Suitable for cardiovascular patches and vascular grafts

**Table 3:** Summary of research in the field of vascular tissue engineering involving amniotic fluid stem cells (AFSCs), amniotic ECs (AECs), and amniotic mesenchymal stem cells (AMSCs)

	Cell Type/Source	Studies Features/Application	Details of Results	Reference
1	AEC/AMSC	Hemocompatibility	Reduced platelet adhesion and activation	[51]
2	AEC	Tumor Vascular Integration	AECs integrate into the tumor vascular lumen; and differentiate into ECs or smooth muscle cells	[45]
3	AFSC	Synergistic Effect on Vascular Networks	Coculture with AFSC-EC in fibrin/PEG matrix enhances network parameters	[48]
4	AFSC	GDNF Impact on Differentiation	GDNF enhances differentiation into endothelial-like cells; expression of vWF and CD31 after 1 week	[47]
5	AFSC	Endothelial Marker Expression	Expression of CD44, CD73, CD90, and HLA-ABC; absence of CD31, CD117, anti-human fibroblasts, HLA-DR, CD34, and CD45	[50]
6	AFSC	Transcription Factor Network	Involvement in Ets and Sox17 TF network for EC fate and function	[49]
7	AFSC	Wide Differentiation Spectrum	The broad range of differentiation capabilities in an ovine model	[52]

### Chorion

The chorion, which lines the inner surface of the trophoblast, is formed from the embryonic mesoderm on the 14th day of human pregnancy, and at this stage, the extracellular space separates it from the amniotic membrane [53].

#### Chorionic membrane

The chorionic membrane serves as the connection between the fetus and maternal tissue and is separated from the amniotic membrane by a layer of collagen fibers. Comprising two layers, the mesenchymal layer and the trophoblastic layer, the chorionic membrane contains MSCs. These cells can differentiate into all three germ layers: mesodermal, endodermal, and ectodermal [54]. Due to its exceptional biomechanical properties and low immunogenicity, the chorionic membrane can be used as a scaffold either independently or in conjunction with an AM for vascular tissue engineering. In the amniochorionic membrane, the epithelial cells of the amniotic membrane and the trophoblast layer of the chorion can be separated and removed through enzymatic solutions and mechanical scraping, resulting in a membrane that supports the growth of ECs and promotes angiogenesis. This unique structure holds significant promise for applications in vascular tissue engineering [55].

#### Chorionic villi

Chorionic villi are finger-like projections on the chorion that harbor cells with a multipotent mesenchymal stromal phenotype. Under suitable induction conditions, these cells can differentiate into various cell types, including neurons, cartilage, osteocytes, and adipocytes [56, 57]. Elevated levels of hydrogen peroxide in cardiovascular diseases and diabetes can lead to endothelial dysfunction, resulting in increased side effects and immune responses and ultimately the onset of thrombosis and atherosclerosis. Mesenchymal stem/stromal cells derived from the chorionic villi of human term placentae (v-MSCs) are able to protect human ECs against hydrogen peroxide-induced damage. v-MSCs reduce the detrimental effects of hydrogen peroxide on the proliferation, migration, angiogenesis, and permeability of ECs. Additionally, they modulate the expression of genes associated with EC cell function, including those related to survival and apoptosis. These cells hold promise for cell therapy aimed at repairing vascular damage caused by glucose and hydroxide peroxide, thereby reducing the adverse effects of cardiovascular diseases and diabetes [58].

Using the principle of tissue engineering techniques in conjunction with autologous cells and biodegradable scaffolds opens up possibilities for the fabrication of heart valves, blood vessels, and myocardial structures. Currently, cardiovascular tissue engineering can be a breakthrough for revolutionizing the treatment of congenital heart diseases, particularly among young patients. A significant challenge in treating these individuals is the absence of a suitable replacement structure similar to a cardiovascular valve. The development of a living and growing autologous structure could eliminate a major obstacle in their treatment journey. Advances in high-resolution imaging technologies now enable the early detection of most defects before birth, typically around the 20th week of gestation. To facilitate treatment, it becomes crucial to isolate cells during pregnancy for the subsequent production of tissue engineering implants either at birth or prenatally.

vMSCs hold promise as a suitable cell source for pediatric tissue engineering applications. Schmidt et al. were the first to suggest the possibility of using these cells for in vitro production of living heart valves [59]. In line with this, a study conducted by Weber et al. involved the extraction of MSCs from chorionic villi that are normally sampled from the applicant and cultivated in vitro. The cells were seeded onto a synthetic biodegradable scaffold to produce heart valve leaflets. The surface of this engineered structure was endothelialized with autologous endothelial progenitor cells derived from amniotic fluid or umbilical cord blood during pregnancy. The resulting tissue exhibited cellular phenotypes, an extracellular matrix composition, and a DNA content comparable to that of its native counterpart. vMSCs are significant for advancing congenital tissue engineering approaches [60].

vMSCs have also shown promise in the treatment of acute myocardial infarction, one of the most critical heart diseases. It has been proven that first-trimester placental chorion mesenchymal stem cells (fCMSCs) are better at reducing the symptoms of this disease in vivo than third-trimester placental chorion mesenchymal stem cells (tCMSCs) and BM-MSCs. Compared with tCMSCs, fCMSCs express higher levels of proangiogenic genes (PDGFD, VEGFA, and TNC), leading to significantly enhanced tube formation. Moreover, fCMSCs exhibit significantly lower expression of antiangiogenic genes (SPRY1 and ANGPTL1) than tCMSCs. Additionally, the improvement in cardiac function achieved by fCMSCs is significantly greater than that achieved by treatment with both tCMSCs and BM-MSCs [61].

#### Chorionic plate

The chorionic plate is composed of the amniochorionic membrane and fetal vessels. As the amniotic membrane is removed, the stem cells of the chorionic plate can be isolated from the area nearest to the umbilical cord. These cells have a mesenchymal phenotype and are able to differentiate into various lineages, including liver, cartilage, adipose, and bone lineages [62]. The decellularized human chorionic plate holds promise for use as a grafting material and in experimental vascularization studies. Cryopreserved human chorionic plate tissue can be decellularized using a perfusion method involving freeze‒thaw steps and chemical treatments. This approach minimizes the use of chemicals and exposure time. In terms of biocompatibility, when primary human umbilical vein endothelial cells (HUVECs) are cultured on this graft, they demonstrate endothelialization. Furthermore, the structure maintains cell-specific phenotypic and expression patterns [63].

The route of cell transplantation to damaged tissue is another critical factor in stem cell therapy. Various methods are available for delivering cells to the heart, including intravenous, intracoronary, coronary sinus, and direct epicardial injection. Among these methods, the direct intramuscular injection method is the most effective in terms of the quantity of delivered cells, with approximately 11% of the transferred cells engrafting into the desired area [64]. Inflammatory cytokines also play a role in the homing and engraftment of stem cells to damaged tissues and organs [65]. Once they reach the target area and are successfully transplanted, these cells can carry out their biological activities, such as proliferation, differentiation, and apoptosis, to facilitate the regeneration of damaged tissue [66].

Jung et al. conducted an in vivo study in rats using labeled chorionic plate-derived MSCs (CP-MSCs) to investigate the optimal transplantation route for treating injured livers. They compared direct transplantation, intrasplenic transplantation, and intravenous transplantation via the tail vein (TTP) with a nontransplanted treatment group and concluded that the therapeutic efficacy of the first two methods was superior to that of the TTP group. This outcome underscores the therapeutic ability of CP-MSCs to promote functional recovery of injured tissues and highlights the importance of selecting the appropriate transplantation routes for achieving the best outcome [67].

Identifying the optimal transplantation route for effective homing of CP-MSCs in cardiovascular diseases remains a significant challenge. Additionally, there is a need to optimize the delivery method to enhance cell engraftment and the survival rate.

Recent studies have shown the advantages of cells derived from the chorionic plate over those derived from the chorionic villi, including greater clonogenic potential and greater expression of cell cycle-related genes, ultimately leading to improved heart function in MI mouse models. Additionally, the exosomes obtained from the culture of these cells show potent angiogenic properties, suggesting that they have valuable potential in vascular tissue engineering [68, 69].

In another study, a comparative analysis involving CP-MSCs, ChorionicVilli-MSCs, decidua-derived MSCs, and umbilical cord blood (UCB) MSCs was conducted, focusing on cell proliferation and migration abilities. The findings showed that CP-MSCs had superior performance in these criteria compared to the other cell types. Additionally, CP-MSCs outperformed other groups in regulating macrophage polarization, specifically shifting macrophages from the M1 phenotype to the M2 phenotype [62]. Notably, compared with CD106-MSCs, CP-MSCs express CD106 markers, which are associated with heightened proliferative capacity and immune regulation potential [70].

### Umbilical cord

The umbilical cord (UC), once considered a biological waste at the time of birth, is now recognized as a perinatal organ that plays a crucial role in facilitating the exchange of nutrients and gases (oxygen and carbon dioxide) between the placenta and the fetus. This remarkable organ, with an average length of 50–60 cm and more than 40 L of blood flow, has emerged as a valuable source of HSCs and MSCs. Importantly, it offers the advantage of being noninvasive and without any ethical concerns. An anatomical cross-section of the umbilical cord revealed that the UC is composed of two arteries and a vein without any branches [71], which are surrounded by Wharton’s jelly, a gelatinous substance rich in ECM proteins, including collagens, glycosaminoglycans such as hyaluronic acid and chondroitin sulfate, as well as growth factors such as IGF-1 and PDGF [72]. Wharton’s jelly serves the vital function of preventing blood vessels from clumping and provides flexibility to the cord. The umbilical cord is further enveloped by a membrane consisting of two layers: mesenchymal and epithelial layers.

Umbilical cord stem cells are a rich source of various stem cell types, including endothelial progenitors, epithelial stem cells, MSCs, and HSCs. These stem cells can be isolated from both cord blood and cord tissue and have shown promise in tissue repair through two primary mechanisms: the release of related cytokines and differentiation into specific cell types needed for tissue regeneration [71].

MSCs derived from Wharton’s jelly (WJ-MSCs) offer several advantages over other sources of MSCs, such as those from the BM. These advantages include a high proliferation rate, a significant capacity for differentiation into various cell types, low immunogenicity, ease of harvesting, and the potential to obtain large quantities of cells during the harvest process [71].

WJ-MSCs secrete various growth factors and cytokines, including G-CSF, HGF, PDGFAA, TGF-β, IL-6, and IL-8. These secreted factors play crucial roles in immunomodulation, cell proliferation, differentiation, growth, and tissue repair, making WJ-MSCs valuable for clinical applications [71, 73].

One notable characteristic of WJ-MSCs is their expression of self-renewal and pluripotency markers, including Oct-4, Sox-2, Nanog, SSEA-4, Tra-1-60, and Tra-1-81. These markers indicate the ability of these cells to maintain their undifferentiated state and their potential to differentiate into various cell lineages [74]. Although MSCs can be successfully isolated from Wharton’s jelly (WJ), the perivascular space (PRV) and the umbilical membrane (UCM) in UC differ, and they may exhibit variations depending on the specific region of isolation [73].

The feasibility of using WJ-MSCs as a suitable cell source for cardiovascular tissue engineering was first demonstrated in 2002. Hoerstrup et al. seeded WJ-MSCs onto a bioabsorbable scaffold in a biomimetic flow culture system and demonstrated the feasibility of generating pulmonary artery conduits using WJ-MSCs. Morphological and mechanical analysis revealed that tissue-engineered pulmonary conduits closely resembled native human pulmonary arteries. This research highlighted the potential of human WJ-MSCs as a readily available cell source for tissue engineering applications, eliminating the need to sacrifice intact vascular donor structures [75].

A more recent study conducted in 2020 investigated the ability of WJ-MSCs seeded on chitosan/hyaluronic acid multilayered films to differentiate into endothelial-like cells. The results were promising, indicating that WJ-MSCs could yield endothelial-like cells in a relatively short time (15 days) in a nontraumatic manner. Such polyelectrolyte films containing an endothelium resulting from the differentiation of MSCs can be used to reduce the risk of graft rejection [76].

Investigating the synergistic effects of gene and stem cell-based therapy on preventing neointimal formation, a common issue associated with vein graft failure, was first performed by Qingxi Qu et al. In this study, the miRNA-126-3p gene, an EC-specific angiogenic miRNA, was transfected into human UC-MSCs using a lentiviral vector. Therapeutic upregulation of miRNA-126-3p had several positive effects, including preventing restenosis in vein grafts, improving EC function through paracrine mechanisms, repairing dysfunctional endothelium, and reducing neointimal hyperplasia in vein grafts in rats. This improvement was attributed to the synthesis and secretion of various bioactive molecules, such as angiogenic factors, growth factors, and cytokines, by MSCs [77].

Severe human disorders, such as cardiovascular and peripheral vascular disease, often require the use of vascular grafts. However, the use of autologous vessels such as the saphenous vein of glutaraldehyde-fixed bovine and porcine xenografts is associated with issues such as a limited number of suitable patient vessels and susceptibility to calcification and chronic immune rejection. One suggested approach is the decellularization of human umbilical arteries with inner diameters ranging from 1 to 4 mm. While decellularization removes cellular components and donor antigens, potentially reducing the risk of immune responses, it leaves behind acellular vascular grafts that need to be repopulated with vascular cell populations to become fully functional. In 2018, an efficient method was developed to repopulate decellularized human umbilical arteries (hUAs) with WJ-MSCs. This approach aimed to produce HLA-matched vascular grafts, addressing some of the limitations associated with conventional graft sources [78]. Vessel bioreactors are commonly used for repopulating vascular grafts under specific conditions, but they often face challenges related to low repopulation efficacy. In an investigation, researchers sought to enhance the repopulation process by culturing human umbilical cord mesenchymal stem cells (hUCMSCs) on decellularized human umbilical arteries in media supplemented with cord blood platelet lysate (CBPL). Mallis et al. reported that CBPL significantly improved cell adhesion, proliferation, and differentiation through the presence of various growth factors, including TGF-β1, FGF, TNF-α, IL-1, IL-3, IL-6, PDGF, and matrix metalloproteases. Moreover, the use of CBPL enabled the differentiation of VSMCs from WJ-MSCs [79]. In another study by Mallis and colleagues, this approach successfully repopulated decellularized hUAs, leading to an increase in total hydroxyproline and sGAG contents. These results suggest that in vitro production of VSMCs, which involves collagen and sGAG synthesis, may be facilitated by factors such as SOX9, RUNX2, and MSX2 [80].

The angiogenic effect of MSCs such as WJ-MSCs on HUVECs has been demonstrated in numerous studies. These effects involve stabilizing the EC network and secreting vasculogenic growth factors, including hepatocyte growth factor. In both autogenic and allogenic cell sources, WJ-MSCs have been shown to promote angiogenesis and enhance vascular tube formation when cocultured with ECs for three days [81].

To ensure the success of endothelialization, it is essential to address challenges such as cell coverage loss due to exposure to physiological levels of shear stress in an active environment. Research has shown that shear stress preconditioning can improve cell retention and enhance the performance of ECs. In one study, HUVECs were preconditioned with shear stress on silk fibroin nanofibrous scaffolds at various time intervals and amplitudes. The results of this research suggest that ECs require sufficient time to acclimate to changing shear stress levels to withstand physiological levels. A gradual increase in shear stress over time can improve EC tolerance to shear stress and enhance the antithrombogenic function of engineered vascular grafts. This process involves an extracellular matrix (ECM)-specific mechanosensitive signaling pathway in which integrin β1, focal adhesion kinase (FAK), and fibronectin (FN) play significant roles [82].

ECs were used to form a monolayer of cells in the lumen of a vascular graft made of polycaprolactone/gelatin/fibrinogen, which was modified by a thermoforming process and coated with fibronectin and collagen IV. The results of this study showed that human cord blood-derived endothelial cells (hCB-ECs) can proliferate, produce endothelial nitric oxide synthase (eNOS), respond to interleukin 1β through the upregulation of VCAM-1 and ICAM-1, and reduce platelet deposition [83].

Expanding available resources for autologous to allogeneic (analog) applications in vascular tissue engineering faces a significant challenge due to the expression of human leukocyte antigen class I (HLA I) on the cell surface of ECs, which can lead to antibody-mediated immune responses and graft rejection. Recent advances in the field have used RNA interference (RNAi) to stably silence the expression of HLA I proteins in ECs via lentiviral vectors without affecting the morphological and functional properties of ECs. The silenced ECs were able to maintain the expression of key endothelial markers, including endothelial nitric oxide synthase, von Willebrand factor, CD31, and vascular endothelial cadherin. These markers are essential for maintaining a functional endothelial barrier, regulating blood coagulation, and controlling vessel tone.

Furthermore, HLA I-silenced ECs retained their ability to perform crucial endothelial functions. They are capable of absorbing acetylated low-density lipoprotein (acLDL) and forming capillary-like tube structures when embedded in 3D fibrin gels and exposed to unidirectional flow, similar to nontransduced cells [84]. The proliferation of ECs is aided by sphingosine-1-phosphate (S1P), an effective additive that also protects Syndecan-1 (SDC1) from shedding, which is important for preventing platelet adhesion. Compared to the controls, their results demonstrated that S1P reduced thrombus formation and enhanced HUVEC proliferation [85].

From another point of view, the physiological characteristics of conduit endothelialization strategies are inferior to those of native vessels due to the absence of VSMCs, which play a crucial role in vessel structure and function [86]. As an example of the utilization of smooth muscle cells in vessel engineering, human umbilical artery-derived smooth muscle cells (hUASMCs) were cultured on an electrospun scaffold containing fibrinogen extracted from human umbilical cord blood samples. To enhance the poor elastogenesis property of the scaffold, PCL was incorporated as a reinforcing material [87].

In 2018, Gökçinar-Yagci and her colleagues attempted to create a fully natural triple-layered vascular construct that mimics all the layers of a blood vessel using natural scaffolds and differentiated vascular cells. In this study, a triple-layered vascular construct was created by combining SMCs and fibroblasts, which were differentiated from perivascular cells (PCs) extracted from the human umbilical cord vein. Collagen type I/elastin/dermatan sulfate was used to form the tunica media, and collagen type I/fibrin was used for the tunica adventitia. Subsequently, HUVECs were seeded onto the construct using the cell sheet engineering method [88].

While this vascular graft closely resembled native blood vessels with a diameter of less than 5 millimeters, its weak mechanical properties made it unable to withstand the pressure within a blood vessel. To address this limitation, another study introduced a biocompatible electrospun polyurethane (PU) scaffold between the tunica intima and media layers of the construct to provide mechanical reinforcement. Polyurethane nanofibers create a suitable environment for HUVECs, enhancing their tensile strength and elastic modulus. As a result, layers of HUVECs, SMCs, and fibroblasts align with each other, strengthening the graft [89]. An overview of research in the field of vascular tissue engineering involving cells isolated from the umbilical cord is shown in Table 4.

**Table 4:** Summary of research in the field of vascular tissue engineering that involves cells isolated from the umbilical cord

*N*.O.	Cell	Tissue or Scaffold	Result	Reference
1	MSC	Decellularized aortic scaffold	miR503 and miR-222-5p regulated the differentiation of MSC into SMCs.	[90]
2	hucMSCs	Rat vein graft	miRNA3-126-p overexpression accelerates reendothelialization.	[77]
3	ECs	chitosan/hyaluronic acid (HA/CHI) multilayers	lentiviral vector through RNAi has suppressed the HLA I complex in ECs from peripheral blood, umbilical cord blood- and veins.	[84]
4	perivascular cells HUCV	collagen type I/elastin/dermatan sulfate and collagen type I/fibrin	perivascular cells (PCs) were differentiated to SMCs and seeded on collagen type I/elastin/dermatan sulfate and collagen type I/fibrin to form tunica media and tunica adventitia.	[88]
5	perivascular cells HUCV	ECM/glycosaminoglycans and polyurethane nanofibers	HUVECs, SMCs, and fibroblasts formed layers that aligned with each other on Polyurethane nanofibers under perfusion.	[89]
6	hCB-ECs	gelatin/fibrinogen/polycaprolactone scaffolds	hCB-EC growth and maintenance of their phenotype enhanced by seeding on polycaprolactone/gelatin/fibrinogen scaffolds.	[83]
7	ECFCs	Fibrin hydrogel	The sheets of cord blood-derived ECFCs in vitro and in vivo developed prevascular structures under controlled conditions.	[91]
8	HUVECs	silk fibroin nanofibrous	EC retention improved by gradually increasing shear stress with appropriate time steps resulting in the formation of a complete endothelial-like monolayer.	[82]
9	UCPs	Decellularized swine-derived grafts	The elasticity and rupture strain improved by integrating UCPs into the outer layer of the graft.	[92]
10	HUVECs WJ-MSCs		The coculture of HUVECs and WJMSC can develop VLS after 3 days in an endothelial culture medium.	[81]
11	WJ-MSCs	chitosan/hyaluronic acid	WJ-MSCs differentiated into endothelial-like cells on chitosan/hyaluronic acid (HA/CHI) multilayers.	[76]
12	WJ-MSCs	decellularized hUAs	The cord blood platelet lysate improved the repopulation efficacy.	[79]
13	MSCs derived from WJ	decellularization of the human umbilical arteries	In vitro repopulating decellularized hUA with MSC under static conditions resulted in HLA-matched vascular grafts.	[78]
14	endothelial progenitors, WJ	Umbilical-cord blood vessels	Endothelial progenitor/stem cells were used for engineered vessels in vascular Transplantation.	[93]
15	hUASMCs	polycaprolactone-reinforced porcine blood-derived fibrinogen scaffold	An electrospun scaffold has been successfully produced from pooled plasma isolated from human umbilical cord blood samples seeded with hUASMCs.	[87]
16	HUVEC EPC	decellularized human umbilical vein (DHUV)	S1P enhanced the attachment of HUVEC and EPC and SDC1 shedding from HUVEC also reduced.	[85]
17	MSCs derived from WJ	human umbilical arteries (hUAs)	WJ-MSCs were successfully differentiated to VSMCs and efficient repopulation on decellularized hUAs has been achieved.	[80]
18	adipose tissue-derived stromal cells allogeneic WJ-MSCs	decellularization of pericardial matrices	WJCs evoked immunomodulatory properties and ASCs accelerated remodeling.	[94]
19	human endothelial progenitor cells, umbilical cord-derived mesenchymal stem cells, luminal EC coverage	decellularized bovine carotid arteries	thrombogenicity is greatly reduced as a result of surface modification of the Decellularized Bovine Carotid Vessels by Human Vascular Cells.	[95]
20	MSCs	swine small intestinal submucosa	The engineered graft is composed of homogeneous endothelium, which has been treated with multilayered muscle media.	[86]

### Decidua

The decidua, a tissue in the uterus, is divided into three regions: the basalis, capsularis, and parietalis. The decidua basalis and parietalis are abundant sources of mesenchymal stem cells. These versatile cells can differentiate into various cell types from different germ layers, including lung cells, liver cells, cartilage, adipocytes, neurons, osteoblasts, and skeletal and cardiac myocytes, under laboratory conditions. They have demonstrated effectiveness in the treatment of several diseases, such as breast cancer, multiple sclerosis, and diabetes, and in reducing inflammation in the central nervous system [96–99]. Research has indicated that decidual mesenchymal stem cells (DMSCs) are more readily isolated from tissue and more accessible than bone marrow mesenchymal stem cells (BMSCs). Additionally, metabolomics analysis revealed a significant increase in ornithine metabolism, which is related to angiogenesis, in DMSCs.

In vivo studies have shown that the transplantation of DMSCs into murine models of acute myocardial infarction (MI) leads to a significant increase in neovascularization and cardiac remodeling compared to the transplantation of BMSCs. Consequently, compared with BMSCs, DMSCs exhibit superior efficacy in terms of revascularization and cardiac regeneration following MI [100].

Furthermore, the ability of human decidua basalis mesenchymal stem cells (DBMSCs) to survive and function in an inflammatory environment characterized by a high concentration of lipopolysaccharide has been explored. Inflammatory conditions are known triggers for certain diseases, such as arteriosclerosis. The results of this study indicated that there were no significant differences between the DBMSC-treated group and the control group. This finding suggested that DBMSCs can maintain their activities, including adhesion, proliferation, and migration, even under inflammatory conditions. Therefore, DBMSCs have the potential to be promising candidates for the treatment of inflammatory diseases, including arteriosclerosis [101].

In a comparative study, the biological characteristics of UCMSCs and MSCs derived from decidua parietalis (DP-MSCs) were investigated. Various features, including cell doubling times, colony formation rates, immune phenotypes, differentiation capacity, and the levels of secreted factors, were assessed for both cell types. Despite DP-MSCs exhibiting increased levels of keratinocyte growth factor, vascular endothelial growth factor, and stem cell factor, UCMSCs exhibited increased proliferation and colony formation rates. Moreover, UC-MSCs exhibited a shorter doubling time than DP-MSCs. Additionally, the concentration of basic fibroblast growth factor in the supernatant of UC-MSCs was notably greater than that in the supernatant of DP-MSCs. Based on these findings, researchers have concluded that UC-MSCs have great potential for effective applications in vascular tissue engineering and regenerative medicine [102].

## Potential clinical application

When exploring cell therapy in clinical trials, it’s crucial to choose diseases for treatment that not only match the cell product’s action mechanism and show promise in preclinical studies but also address severe illnesses lacking effective treatments. This approach is primarily aimed at conditions that are both severely debilitating and critically lacking in medical solutions, underlining the importance of targeting diseases with no current therapy standards [103].

In cardiovascular treatments, for instance, non-autologous valve or conduit implants often face issues like obstructive tissue growth and calcification. This has led to a shift towards developing autologous, living tissues engineered in vitro, which can regenerate damaged cardiovascular tissue. Among the promising candidates for vascular diseases, placenta-derived cell therapies such as placenta derived adherent stromal cells (PLX-PAD) and Human Placenta-derived Cells (PDA-002) have shown potential, particularly for treating peripheral arterial disease (PAD). Within this area, patient groups are identified based on several factors, including those impacting enrolment and regulatory considerations. PLX-PAD, for example, is seen as particularly suited for critical limb ischemia patients, who face a dire prognosis including high rates of amputation and mortality, and significantly reduced quality of life. These patients, especially that ineligible for revascularization, have no existing treatment options, marking a significant unmet medical need that PLX-PAD aims to address. Early-phase trials in this patient group have shown promising signs of safety and effectiveness, including pain reduction and improved tissue perfusion, providing insights into potentially effective dosages [103, 104].

Furthermore, WJ-MSCs have been recognized for their clinical potential in vascular tissue engineering, with successful applications in animal models and human trials. For instance, autologous heart valves derived from human WJ-MSCs have been effectively used in sheep, demonstrating comparable functional and structural qualities to native valves. In a study involving patients with acute myocardial infarction, WJ-MSCs were safely administered and showed significant improvements in heart tissue viability and perfusion, positioning them as a viable alternative to traditional stem cell therapies for heart repair [105, 106].

The first human trial to assess the safety of administering UCMSCs through both intra-arterial and intravenous routes in patients with acute ischemic stroke is recruiting. This Phase 1 study has enrolled 14 participants to test the innovative UCMSCs treatment. The trial includes a follow-up period of 12 months and explores a method of administration never before attempted in humans, presenting potential new risks and benefits [107]. In another study, researchers are investigating the effectiveness of a novel combination therapy using intranasal conditioned medium and intraparenchymal transplantation of UCMSCs to stimulate neurogenesis in patients with acute strokes. This trial features three groups: one receiving the combination therapy, a second receiving only UCMSCs, and a third serving as a control group with standard neurological and neurotrophic drugs. The hypothesis is that the combination therapy will most effectively promote neurogenesis in these patients [108]. Additionally, a study is set to explore the safety and feasibility of UCMSC catheter transplantation for treating left ventricular dysfunction post-acute myocardial infarction. Slated to start in December 2023 and projected to end in December 2025, this Phase 1 trial will involve 40 patients split into two groups: one receiving the UC-MSC transplantation and the other receiving standard care. The primary focus will be monitoring Major Adverse Cardiac Events (MACE) to evaluate safety during the 12 months following treatment [109].The advancements in using extra-embryonic Stem Cells in regenerative medicine highlight the diverse therapeutic possibilities these cells might offer. Despite the promise, the outcomes from both pre-clinical and clinical studies are still in early stages and require further clarification. The characteristics and therapeutic potential of extra-embryonic MSCs are not fully understood, necessitating additional research into their role in cell therapy and tissue engineering. Additionally, developing biomaterials compatible with these stem cells, without compromising their regenerative and immune-modulating capabilities, remains a critical area for future research [110]. Table 5 shows the clinical approaches using extra-embryonic stem cells.

**Table 5:** Summary of research using extra-embryonic stem cells in clinical application

Number	Cell Type	Description	Reference
1	PLX-PAD, PDA-002	Used in treating peripheral arterial disease (PAD), particularly suitable for critical limb ischemia patients. Early-phase trials show promise in pain reduction and improved tissue perfusion.	[103, 104]
2	WJ-MSCs	Shown potential in vascular tissue engineering; used in animal models and human trials. Demonstrated significant improvements in heart tissue viability and perfusion post-acute myocardial infarction. Autologous heart valves from human WJ-MSCs used effectively in sheep.	[105, 106]
3	UCMSCs	First human trial via intra-arterial and intravenous routes for acute ischemic stroke; exploring novel administration methods. Also investigated for stimulating neurogenesis in acute stroke patients using a combination therapy of intranasal conditioned medium and intraparenchymal transplantation. A separate study explores catheter transplantation for left ventricular dysfunction post-acute myocardial infarction.	[107–109]

## Conclusion

Vascular tissue engineering is a promising approach for regenerating damaged blood vessels and developing new therapeutic strategies for heart diseases. Perinatal stem cells, derived from extraembryonic tissues, have immense potential in addressing the symptoms of cardiovascular diseases due to their angiogenic, immunomodulatory, and endothelialization-promoting properties. Recent studies suggest that not only the perinatal stem cells themselves but also the soluble and insoluble factors they release could play a pivotal role in tissue regeneration. Soluble factors like growth factors (e.g., VEGF, FGF), cytokines (e.g., IL-6, TNF-alpha), and hormones (human Chorionic Gonadotropin, human Placental Lactogen) alongside insoluble factors such as, collagen and fibronectin, may offer a cell-free therapeutic approach. This innovative strategy provides a novel direction for future research that could potentially simplify therapeutic strategies, reduce risks associated with cell-based therapies, and enhance the scalability of treatments. However, more research is required to overcome the existing challenges and to translate these promising findings into effective clinical therapies, which can offer new hope for patients with cardiovascular diseases. In conclusion, the field of vascular tissue engineering, particularly through the innovative use of extraembryonic tissue-derived bioactive factors, is rapidly evolving and holds great promise for the future of cardiovascular disease treatment.

## Data Availability

Not applicable.

## Abbreviations

acLDL: Acetylated low-density lipoprotein
AECs: Amniotic epithelium cells
AF: Amniotic fluid
AFMSCs: Amniotic fluid mesenchymal stem cells
AFSCs: Amniotic fluid stem cells
AFSC-ECs: Amniotic fluid stem cell-derived endothelial cells
AM: Amniotic membrane
AMSCs: Amniotic mesenchymal stem cells
ASCs: Adipose-derived stem cells
BMSCs: Bone marrow mesenchymal stem cells
CaMKIIγ: Ca2+/calmodulin-dependent protein kinase II
CBPL: Cord blood platelet lysate
CT: Clotting time
DMSCs: Decidual mesenchymal stem/stromal cells
DBMSCs: Human Decidual Basalis Mesenchymal Stem Cells
ECs: Endothelial cells
ECM: Extracellular matrix
EPCs: Endothelial progenitor cells
ESCs: Extraembryonic tissue-derived cells
ePTFE: Expanded polytetrafluoroethylene
eNOS: Endothelial nitric oxide synthase
FAK: Focal adhesion kinase
fCMSCs: First-trimester placental chorion mesenchymal stem cells
GDNF: Glial cell line-derived neurotrophic factor
MSC: Mesenchymal stem cell
MI: Myocardial infarction
iPSCs: Induced pluripotent stem cells
HSCs: Hematopoietic stem cells
HUVECs: Human umbilical vein endothelial cells
HLA I: Human leukocyte antigen class I
hUAs: Human umbilical arteries
hCB-ECs: Human cord blood-derived endothelial cells
SMCs: Smooth muscle cells
SMPCs: Smooth muscle progenitor cells
SDF-1: Stromal cell-derived factor 1 receptor
S1P: Sphingosine-1-phosphate
VEGF: Vascular Endothelial Growth Factor
VEGFR1: Vascular Endothelial Growth Factor receptor-1
v-MSCs: Mesenchymal stem/stromal cells derived from chorionic villi
vWb: von Willebrand factor
PCL: Polycaprolactone
PCs: Perivascular cells
PSCs: Perinatal stem cells
PDGF: Platelet-Derived Growth Factor
PDGFR: Platelet-derived growth factor receptor
PGA: Poly glycolic acid
PLA: Polylactic acid
PLX-PAD: Placenta derived adherent stromal cells
PDA-002: Human Placenta-derived Cells
PT: Prothrombin time
PU: Polyurethane
aPTT: Activated partial thromboplastin time
RNAi: RNA interference
TEVGs: Tissue engineering vascular grafts
TGF-β: Transforming growth factorβ
TFs: Transcription factors
tCMSCs: Third-trimester placental chorion mesenchymal stem cells
SPC: Sphingosylphosphorylcholine
SMA: Smooth muscle actin
UC: Umbilical cord
hUC: Human umbilical cord
UCB: Umbilical cord blood
UCPs: Umbilical cord pericytes
UCMSCs: Umbilical cord mesenchymal stem cells
VSMCs: Vascular smooth muscle cells
WJ-MSCs: Wharton’s jelly mesenchymal stem cells
